# Cellular apoptosis and cell cycle arrest as potential therapeutic targets for eugenol derivatives in *Candida auris*

**DOI:** 10.1371/journal.pone.0285473

**Published:** 2023-06-21

**Authors:** Hammad Alam, Vartika Srivastava, Windy Sekgele, Mohmmad Younus Wani, Abdullah Saad Al-Bogami, Julitha Molepo, Aijaz Ahmad

**Affiliations:** 1 Faculty of Health Sciences, Department of Clinical Microbiology and Infectious Diseases, School of Pathology, University of the Witwatersrand, Johannesburg, South Africa; 2 Faculty of Health Sciences, Department of Oral Biological Sciences, School of Oral Health Sciences, University of the Witwatersrand, Johannesburg, South Africa; 3 Department of Chemistry, College of Science, University of Jeddah, Jeddah, Saudi Arabia; 4 Infection Control, Charlotte Maxeke Johannesburg Academic Hospital, National Health Laboratory Service, Johannesburg, South Africa; University of New South Wales, AUSTRALIA

## Abstract

*Candida auris*, the youngest *Candida* species, is known to cause candidiasis and candidemia in humans and has been related to several hospital outbreaks. Moreover, *Candida auris* infections are largely resistant to the antifungal drugs currently in clinical use, necessitating the development of novel medications and approaches to treat such infections. Following up on our previous studies that demonstrated eugenol tosylate congeners (ETCs) to have antifungal activity, several ETCs (C1-C6) were synthesized to find a lead molecule with the requisite antifungal activity against *C*. *auris*. Preliminary tests, including broth microdilution and the MUSE cell viability assay, identified C5 as the most active derivative, with a MIC value of 0.98 g/mL against all strains tested. Cell count and viability assays further validated the fungicidal activity of C5. Apoptotic indicators, such as phosphatidylserine externalization, DNA fragmentation, mitochondrial depolarization, decreased cytochrome c and oxidase activity and cell death confirmed that C5 caused apoptosis in *C*. *auris* isolates. The low cytotoxicity of C5 further confirmed the safety of using this derivative in future studies. To support the conclusions drawn in this investigation, additional *in vivo* experiments demonstrating the antifungal activity of this lead compound in animal models will be needed.

## Introduction

*Candida auris* was identified as a novel human pathogen in 2009, and it has subsequently caused considerable healthcare problems by producing systemic infections in individuals with underlying diseases [1–3]. Even though *C*. *auris* shares a strong evolutionary relationship with other pathogenic *Candida* species, it differs from them in terms of biology, genetics, epidemiology, antifungal resistance, virulence, host adaptation, and transmission [4,5]. It was recently identified as a critically important fungal pathogen due to its inherent resistance to the majority of presently used antifungal medications, as well as pan-resistance in certain isolates. [6]. Echinocandins are often used to prevent *C*. *auris* infections, particularly in patients in critical care or who have had invasive surgical procedures [7,8]. The high mortality rates associated with this pathogen have been attributed to multidrug resistance, accompanying hospital outbreaks, and invasive infections [9,10]. Another clinically important problem is the misdiagnosis of *C*. *auris*, as laboratory yeast identification methods frequently mistake it with other yeasts [11]. Hence, to combat infections caused by *C*. *auris* and other developing fungal diseases, it is imperative to develop novel, secure, and effective antifungal drugs, and treatment strategies with a range of pharmacological targets.

Natural product-based antifungal medications are typically regarded as being efficient, affordable, and safer with less toxicity [12]. Due to the antibacterial, antiviral, antifungal, anticancer, anti-inflammatory, and antioxidant properties associated with eugenol and its derivatives, they have long been used in cosmetology, medicine, and pharmacology [13,14]. Eugenol and other monoterpene phenols have been demonstrated to inhibit the production of ergosterol and, also inhibit efflux pump inhibitors in *C*. *albicans* and other non-albicans *Candida* species, resulting in the reversal of drug resistance among these pathogens [15]. In our previous studies, derivatization of eugenol led to the development of eugenol tosylates (ETC’s) with improved antifungal efficacy and safety profile than the parent compound eugenol [14,16,17]. In this study different ETCs (C1–C6) were synthesized in a quest to find a lead molecule with desired antifungal activity against *C*. *auris*.

## Materials and methods

The chemical reagents and solvents were procured from Sigma Aldrich and Merck Germany. TLC plates used were precoated aluminium sheets (silica gel 60 F254, Merck Germany) and visualization was done by UV light in a UV cabinet. Heraeus Vario EL III analyser was used for elemental analysis. Bruker ALPHA FT-IR spectrometer (Eco-ATR) was used for FTIR analysis. Bruker AVANCE 400 spectrometer (400 MHz) was used for ^1^H and ^13^C NMR spectra using DMSO-d6/CDCl3 as solvent with TMS (Tetramethylsilane) as standard. ESI-MS positive ion mode was recorded on Micromass Quattro II triple quadrapole mass spectrometer.

### Synthesis

Eugenol, also known as 2-methoxy-4-(prop-2-en-1-yl) phenol, was used as the starting material for all the compounds C1–C6. For their synthesis, eugenol was treated with various phenyl and substituted phenylsulfonyl chlorides in refluxing pyridine for 18–24 hours. (Completion of reaction was monitored by TLC). After the completion of the reaction, the reaction mass was quenched with distilled water and extracted with dichloromethane. Finally, the combined organic layers were washed with distilled water again and dried over anhydrous Na_2_SO_4_. The compounds were further purified using column chromatography using dichloromethane and methanol in the ratio of 7:3 as eluant. The detailed synthesis route has been discussed previously [18].

#### 2-methoxy-4-(prop-2-en-1-yl)phenyl-4-methylbenzenesulfonate (C1)

Yield: 85%; Anal. Calc. for C_17_H_18_O_4_S; C, 64.13; H, 5.70%, found; C, 64.28; H, 5.56%; IR_max_cm^-1^: 3035 (C-H stretch), 1585 (C = C, Ar), 1385, 1155 (S = O); ^1^H NMR (DMSO-*d*_*6*_) (ppm): 7.87–7.59 (4H, m, Ar-H), 6.88–6.78 (3H, m, Ar-H), 6.25 (1H, m), 4.86 (1H, dd, J = 15.2 Hz, 6.8 Hz), 4.48 (1H, dd, J = 15.2 Hz, 6.8 Hz), 3.90 (2H, d, CH_2_), 3.64 (3H, s, OCH_3_), 2.25 (3H, s, CH_3_); ^13^CNMR (DMSO-*d*_*6*_) (ppm): 148.9, 144.6, 138.5, 137.0, 135.4, 133.5, 130.6, 129.3, 128.0, 122.5, 118.5, 117.0, 115.7, 56.4, 47.4, 24.8; ESI-MS m/z [M+H]^+^ 319.10; [M+Na]^+^ 341.08.

#### 2-methoxy-4-(prop-2-en-1-yl)phenyl-4-nitrobenzenesulfonate (C2)

Yield: 89%; Anal. Calc. for C_16_H_15_NO_6_S; C, 55.01; H, 4.33%; N, 4.01; found; C, 55.28; H, 4.30%, N, 4.20; IR_max_cm^-1^: 3035 (C-H stretch), 1582 (C = C, Ar), 1385, 1152 (S = O); ^1^H NMR (DMSO-*d*_*6*_) (ppm): 8.48–8.36 (4H, m, Ar-H), 6.88–6.78 (3H, m, Ar-H), 6.28 (1H, m), 4.97 (1H, dd, J = 15.2 Hz, 6.8 Hz), 4.46 (1H, dd, J = 15.2 Hz, 6.8 Hz), 3.94 (2H, d, CH_2_), 3.40 (3H, s, OCH_3_); ^13^CNMR (DMSO-*d*_*6*_) (ppm): 152.8, 150.5, 141.7, 139.0, 138.0, 135.3, 128.4, 124.7, 122.1, 120.9, 116.4, 112.6, 56.5, 43.0; ESI-MS m/z [M+H]^+^ 350.42; [M+Na]^+^ 372.40.

#### 2-methoxy-4-(prop-2-en-1-yl)phenyl-4-iodobenzenesulfonate (C3)

Yield: 82%; Anal. Calc. for C_16_H_15_IO_4_S; C, 44.67; H, 3.51%, found; C, 44.76; H, 3.62%; IR_max_cm^-1^: 3038 (C-H stretch), 1584 (C = C, Ar), 1384, 1155 (S = O); ^1^H NMR (DMSO-*d*_*6*_) (ppm): 7.64–7.39 (4H, m, Ar-H), 6.99–6.94 (3H, m, Ar-H), 6.25 (1H, m), 4.79 (1H, dd, J = 15.2 Hz, 6.8 Hz), 4.49 (1H, dd, J = 15.2 Hz, 6.8 Hz), 3.90 (2H, d, CH_2_), 3.61 (3H, s, OCH_3_); ^13^CNMR (DMSO-*d*_*6*_) (ppm): 150.9, 142.5, 138.5, 138.0, 135.1, 134.2, 130.7, 123.8, 121.5, 117.5, 112.1, 101.9, 55.8, 40.3; ESI-MS m/z [M+H]^+^ 331.30; [M+Na]^+^ 345.26.

#### 2-methoxy-4-(prop-2-en-1-yl)phenyl-4-bromobenzenesulfonate (C4)

Yield: 80%; Anal. Calc. for C_16_H_15_BrO_4_S; C, 50.14; H, 3.95%, found; C, 55.25; H, 4.05%; IR_max_cm^-1^: 3034 (C-H stretch), 1585 (C = C, Ar), 1386, 1158 (S = O); ^1^H NMR (DMSO-*d*_*6*_) (ppm): 7.84–7.68 (4H, m, Ar-H), 7.00–6.80 (3H, m, Ar-H), 6.21 (1H, m), 4.83 (1H, dd, J = 15.2 Hz, 6.8 Hz), 4.45 (1H, dd, J = 15.2 Hz, 6.8 Hz), 3.87 (2H, d, CH_2_), 3.64 (3H, s, OCH_3_); ^13^CNMR (DMSO-*d*_*6*_) (ppm): 150.4, 139.6, 137.8, 136.9, 134.8, 133.7, 129.5, 122.8, 119.9, 116.4, 112.6, 56.5, 44.9; ESI-MS m/z [M+H]^+^ 384.26; [M+Na]^+^ 406.30.

#### 2-methoxy-4-(prop-2-en-1-yl)phenyl-4-(bromomethyl)benzenesulfonate (C5)

Yield: 70%; Anal. Calc. for C_17_H_17_O_4_S; C, 64.13; H, 5.70%, found; C, 64.28; H, 5.56%; IR_max_cm^-1^: 3035 (C-H stretch), 1585 (C = C, Ar), 1387, 1155 (S = O); ^1^H NMR (DMSO-*d*_*6*_) (ppm): 7.84–7.68 (4H, m, Ar-H), 7.00–6.80 (3H, m, Ar-H), 6.21 (1H, m), 4.83 (1H, dd, J = 15.2 Hz, 6.8 Hz), 4.46 (1H, dd, J = 15.2 Hz, 6.8 Hz), 4.20 (2H, s, CH_2_), 3.87 (2H, d, CH_2_), 3.64 (3H, s, OCH_3_); ^13^CNMR (DMSO-*d*_*6*_) (ppm): 150.6, 143.2, 138.8, 138.0, 136.7, 133.8, 130.6, 127.8, 123.5, 123.0, 117.2, 113.7, 56.4, 42.5, 34.0; ESI-MS m/z [M+H]^+^ 398.30; [M+Na]^+^ 420.40.

#### 2-methoxy-4-(prop-2-en-1-yl)phenyl-4-propylbenzenesulfonate (C6)

Yield: 75%; Anal. Calc. for C_19_H_22_O_4_S; C, 65.87; H, 6.40%, found; C, 65.95; H, 6.48%; IR_max_cm^-1^: 3036 (C-H stretch), 1587 (C = C, Ar), 1385, 1156 (S = O); ^1^H NMR (DMSO-*d*_*6*_) (ppm): 7.80–7.54 (4H, m, Ar-H), 6.68–6.57 (3H, m, Ar-H), 6.25 (1H, m), 4.83 (1H, dd, J = 15.2 Hz, 6.8 Hz), 4.57 (1H, dd, J = 15.2 Hz, 6.8 Hz), 3.91 (2H, d, CH_2_), 3.61 (3H, s, OCH_3_), 2.65 (2H, t, CH_2_), 1.63 (2H, m, CH_2_), 1.11 (3H, t, CH_3_); ^13^CNMR (DMSO-*d*_*6*_) (ppm): 151.0, 146.1, 139.5, 138.0, 135.4, 132.6, 128.8, 126.6, 121.4, 120.1, 115.0, 112.8, 56.5, 42.7, 36.7, 24.3, 13.8; ESI-MS m/z [M+H]^+^ 347.45; [M+Na]^+^ 369.46.

### Ethics statement

All the *Candida auris* isolates (n = 5) were obtained from the Division of Mycology, National Institute of Communicable Diseases (NICD), Johannesburg, South Africa. To use these isolates in this study, an ethics waiver was obtained from the Human Research Ethics Committee of University of the Witwatersrand (M140159) and performed according to guidelines outlined in the Helsinki Declaration. All the isolates were revived on Sabouraud Dextrose Agar (SDA) before the experiments.

### Antifungal activity of eugenol derivatives (C1–C6)

Antifungal activity of the newly synthesized compounds was done against *C*. *auris* isolates following Clinical and Laboratory Standards Institute (CLSI) recommendations M27-A3 guidelines [19]. The stock concentrations of all the test congeners were prepared to 5000 μg/ml using 1% DMSO, leading to the final test concentrations ranged from 1250–11.34 μg/ml after serial dilution. After incubation at 37°C for 24 hours MIC values were visually observed as the lowermost concentrations of the test compounds (C1–C6) at which no fungal growth was seen. Amphotericin B and 1% DMSO were used as positive and negative controls in the susceptibility assays.

To further determine MFC, all wells that showed no growth were sub-cultured on SDA plates. After incubation at 37°C for 24 hours, MFC were recorded as the least concentrations of test congeners that resulted in total fungal mortality (99.9%).

### Cell viability assay

In presence or absence of the active compound (C5) the cell viability of *C*. *auris* 6057 was checked with the Guava® Muse® Cell Analyzer following manufacturer’s instructions. Briefly, for cell viability *C*. *auris* cells were grown till log-phase and then centrifuged for 5 minutes at 4000 g. The cells were then washed three times with phosphate buffer saline (PBS), followed by exposing with the different concentrations (½ MIC, MIC, 2MIC) of C-5 at 37°C for 4 hours. After incubation cells were washed and resuspended in PBS. Cell viability was quantified by adding 20 μL of cell suspension and 380 μL of count & viability reagents at room temperature for 5 minutes in dark. The results were calculated by reading cell count and cell viability using the Guava® Muse® Cell Analyzer.

### Apoptotic studies

#### Protoplast preparation

The apoptotic studies were done by making the protoplast of *C*. *auris* healthy or C5 treated cells. Protoplasts were made from *C*. *auris* MRL6057 cells using the previously published protocol [18].

#### Effect of C5 on membrane potential of mitochondria (Δψm)

The effect of the most active compound C5 on *C*. *auris* mitochondrial membrane potential (MMP Δψm) was quantified by using the JC-10 kit (Abcam, UK), according to the directions of manufacture. Briefly, 90 μL of the prepared protoplasts were added with 50 μL JC-10 dye into vibrant bottom and blackened walled 96-well plates and left in dark at room temperature for 1 hour. After incubation period, 50 μL volume of kit’s buffer-B was added, and then microtiter plate was rotated at 800g for 2 minutes. The excitation-emission maxima ratio (Ex/Em = 490/530nm and 540/590nm) was calculated using a microplate reader (Spectra-Max iD-3 multi-mode, USA). The fluorescence intensity (green fluorescence) referred to as X was calculated by using Ex/Em = 490/530nm, while the red fluorescence referred to as Y was calculated by using Ex/Em = 540/590nm. The reduction in mitochondrial membrane potential (MMP) in exposed cells was measured using the aggregate/monomeric (Y-mean/X-mean) ratio of JC10 dye. The decrease in ratio was regarded as depolarization of the mitochondrial membrane. During the tests, negative (untreated cells) and positive (treated with 10 mM H_2_O_2_) controls were also included.

#### Effect on *C*. *auris* cytochrome c oxidase discharge

Using a previously described protocol, the effect of a C5 on cytochrome c oxidase release in *C*. *auris* was studied [20]. Briefly 1×10^6^ CFU/mL *C*. *auris* cells were exposed with different concentrations (½ MIC, MIC and 2 MIC) of compound C5 for 4 hours at 37°C with shaking. The positive control (10 mM H_2_O_2_ treated cells) and the negative control (untreated *C*. cells) were also included. After exposure to test compounds cells were harvested, rinsed with PBS, and then homogenized in buffer-A (EDTA 1mM, phenylmethylsulfonyl fluoride (PMSF) 1mM, tris base 50mM, 7.5pH). After homogenization cells were subjected to another cycle of centrifugation at 4000g for 10 minutes. The supernatant was collected separately in microcentrifuge tubes and centrifuged at 15,000 g for 45 minutes. The cell free suspension was pipetted out in microcentrifuge tubes and used to calculate the cytochrome c concentration in the cytoplasm. Meanwhile, the pellet was dispersed in buffer-B (EDTA 2mM, tris base 50mM; 5.0pH) and used to calculate the quantity of cytochrome c in mitochondria. Before taking absorbance ascorbic acid (500 mg/mL) was added in 1:1 ratio, to calculate the amount of cytochrome c in mitochondria and cytosol by taking absorbance at 550 nm with a UV-1800 SHIMADZU spectrophotometer.

#### DNA disintegration analysis by TUNEL-assay

The TUNEL assay, also referred to as terminal deoxy-nucleotidyl transferase dUTP nick end labelling staining, is used to identify DNA breaks or fragmentations produced during the last stages of apoptosis. For the TUNEL assay, overnight grown *C*. *auris* cells were collected and resuspended in PBS *followed by* treatment with ½MIC, MIC, and MFC values of the compound C5. H_2_O_2_ treated *C*. *auris* cells acts as the positive control and healthy cells serve as the negative control. The cells were rinsed three times with PBS and fixed in fixative solution (4% paraformaldehyde) for 30 minutes. The cells were put in 0.25% triton X-100 for 2 minutes for permeabilization. After permeabilization the *C*. *auris* cells were washed with PBS and incubated with Click-iT Plus TUNEL-assay kit (Thermo Fisher Scientific’s) for 30 minutes in the dark. Additionally, *Candida* cells were stained with 50μl of Hoechst 33342 (1X) dye (Thermo-Fisher Scientific USA) and left-over to sit for 15 minutes in the dark at room temperature, and cell were washed two time with PBS, before going to observe under the Confocal Microscopy (Zeiss Laser-Scanning Confocal-Microscope (LSM) 780 Jena, Germany). The excitation-wavelength of 495nm and an emission-wavelength of 519nm for the Click-iT Plus TUNEL assay dye and for Hoechst-33342 dye had an excitation wavelength of 350nm and an emission wavelength of 461nm was used.

#### Cell cycle arrest

The cell cycle arrest is a point in the cell cycle at which cells no longer participate in the processes of cell duplication and division. The effect of C5 on the cell cycle was determined by allowing yeast cells to grow for 8 hours, harvested at 4000g followed by suspension of 0.5 McFarland cells in SD broth. Different concentrations of C5 (½ MIC, MIC, and 2MIC) was added to different tubes followed by incubation for 4 hours with shaking. After, incubation the protoplasts were prepared according to protocol as above describe. The fixed cells were then incubated with the Muse^TM^ Cell Cycle reagents, which contains ribonuclease and propidium iodide for 30 minutes and cell cycle analysis was determined using Muse® Cell Analyzer. The percentage of dead and live cells in each phase of the cell cycle was then visualised.

#### Cytotoxicity of C5 compound

The cytotoxic potential of C5 was assessed using horse red blood cells (purchased from National Health Laboratory Service, Johannesburg, South Africa) following a previously described method. [18]. Briefly, 50ml of sterile falcon tubes containing 10 mL of horse blood were spun for 10 minutes at 2000rpm. The resultant red blood cells (RBSs) pellet was thoroughly washed three times with a cooled PBS solution before the cells were suspended once more in a cold PBS solution to produce a 10% RBC suspension. This RBC sample was once more diluted with a PBS solution ten times. As a result, a positive control and three different C5 compound concentrations (½MIC, MIC, and MFC) were added to the resulting 1mL RBC suspension. The treated RBC suspension was then maintained at room temperature for 1 hour and then centrifuged for 10 minutes at 2000rpm. 200μL supernatant was aliquoted in flatbottom 96-well plate (Thermo Fisher Scientific, Germany), and absorbance was measured at 450nm with SpectraMax iD3 multi-mode microplate reader. In this experiment Triton X 100 (1%) was kept as positive control and whereas, the fresh PBS as negative control. According to Lone et al., the percent hemolysis was calculated [18].

## Results and discussion

### Chemistry

**Fig 1:**
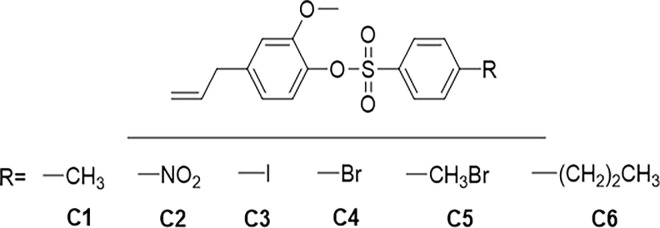
Scheme 1: Structures of derivatives C1-C6.

Eugenol (EUG) or 4-allyl-2-methoxyphenol is a naturally occurring compound that is known to show antifungal properties [21]. Eugenol has been known to scavenge free radicals, inhibit the generation of reactive oxygen species and increase cyto-antioxidant potential. Besides it is also known to increase H_2_O_2_ and Ca^2+^ concentrations in the cytoplasm, which links the antifungal activity of this compound to plasma membrane damaging and destabilizing properties [22]. Despite having potent antifungal properties, eugenol has also been found to show hepatotoxicity, contact stomatitis, and allergic cheilitis besides other complications. It also displays low chemical stability and is sensitive to oxidation and various chemical interactions [13]. To mitigate these side effects and to improve the biological profile of this molecules, various modifications of the structure of this molecule have been carried out [13,23]. In a previous study, it was reported that eugenol tosylate and its congeners, prepared by the functionalization of the hydroxyl group of eugenol with different sulfonyl chlorides under basic conditions, showed promising antifungal activities compared to eugenol. In this study, more derivatives were synthesized and screened to find a suitable molecule with desired antifungal activity as illustrated in **Scheme 1 (Fig 1)**. Elemental analysis, FTIR, ^1^HNMR, ^13^CNMR, and MS-ESI^+^ spectrum studies were used to determine the structure of the compounds. The synthesis of eugenol-tosylate and its congeners C1-C6 was demonstrated using FTIR spectra. The presence of bands around 1156–1158 cm^-1^ and 1385–1387 cm^-1^ corresponding to the sulfonyl group of the respective derivatives and the absence of any free or H-bonded band at/or about 3200–3700 cm^-1^ corresponding to -OH provides strong proof for the formation of the desired compounds. The absence of any signal for the OH proton in ^1^H and ^13^CNMR, as well as the emergence of distinctive peaks at predicted chemical shifts and integral values for phenyl ring and allyl protons, give strong evidence for the synthesis of the C1-C6 derivatives. All of the derivatives (C1-C6) had a [M+H]^+^ peak in their mass spectra, which corresponded to the chemical formula of the compounds, further verifying their synthesis. The molecular ion peaks in some derivatives were found as [M+Na^+^]^+^ (Metal adduct ions). The physical and spectral data are presented in the experimental section.

### Antifungal activity

*C*. *auris* is a public health concern due to its resistance and proclivity to create nosocomial epidemics. Fungal infections caused by this pathogen have high morbidity and mortality rates. *C*. *auris*, despite being the youngest *Candida* species, is resistant to all major antifungal drug classes [24]. Therefore, there is an inevitable need of developing new antifungal drugs with novel approaches to curb the infections caused by multidrug resistant pathogens like *C*. *auris*. Eugenol, a natural compound from clove essential oil, has been thoroughly studied for its antimicrobial activities [25]. Derivatization of eugenol has also been reported to enhance its antimicrobial properties and safety usage [14]. Modifying natural compounds to improve their efficacy, solubility, and safe use has been appreciated in the discovery of innovative medications against a variety of infectious disorders, including candidiasis [26,27].

### Susceptibility testing

The *in-vitro* susceptibility of six newly synthesized eugenol derivatives (C1–C6) was assessed against 5 different *C*. *auris* strains by measuring minimum inhibitory concentrations (MIC) and minimum fungicidal concentrations (MFC) (Table 1). All the test compounds showed antifungal activity with varying MIC and MFC values against *C*. *auris* isolates with compound C5 being the most potent with MIC and MFC values of 0.98 and 1.95 μg/mL respectively. Regarding the selected *C*. *auris* strains, all the strains except *C*. *auris* 6057 were susceptible to amphotericin B (AmB) which served as a positive control in this study. Interestingly C5 showed equally potent antifungal activity against the amphotericin B susceptible and resistant isolates. Based on MIC and MFC results, C5 showed considerably high anticandidal activity out of the six compounds examined and resistant isolate *C*. *auris* 6057 carried out for further study.

**Table 1:** Minimum inhibitory concentrations (MIC) and minimum fungicidal concentrations (MFC) of eugenol tosylate congeners (C1-C6) against different *C*. *auris* isolates.

Compounds		*C*. *auris* 6005	*C*. *auris* 6015	*C*. *auris* 6057	*C*. *auris* 6059	*C*. *auris* 6065
**C1**	**MIC (μg/mL)**	125	125	>125	125	125
	**MFC (μg/mL)**	>125	>125	>125	>125	>125
**C2**	**MIC (μg/mL)**	31.25	15.62	31.25	15.62	31.25
	**MFC (μg/mL)**	125	62.5	62.5	31.5	62.5
**C3**	**MIC (μg/mL)**	3.91	3.91	3.91	3.91	3.91
	**MFC (μg/mL)**	7.81	7.81	7.81	7.81	7.81
**C4**	**MIC (μg/mL)**	31.25	15.62	31.25	31.25	31.25
	**MFC (μg/mL)**	62.5	62.5	125	31.5	62.5
**C5**	**MIC (μg/mL)**	**0.98**	**0.98**	**0.98**	**0.98**	**0.98**
	**MFC (μg/mL)**	1.8	1.8	1.8	1.8	1.8
**C6**	**MIC (μg/mL)**	125	62.5	62.5	125	62.5
	**MFC (μg/mL)**	>125	>125	>125	>125	>125
**AmB**	**MIC (μg/mL)**	1	0.25	4	0.5	1
	**MFC (μg/mL)**	2	0.5	8	1	2

amphotericin B (AmB)*.

### Cell viability

**Fig 2:**
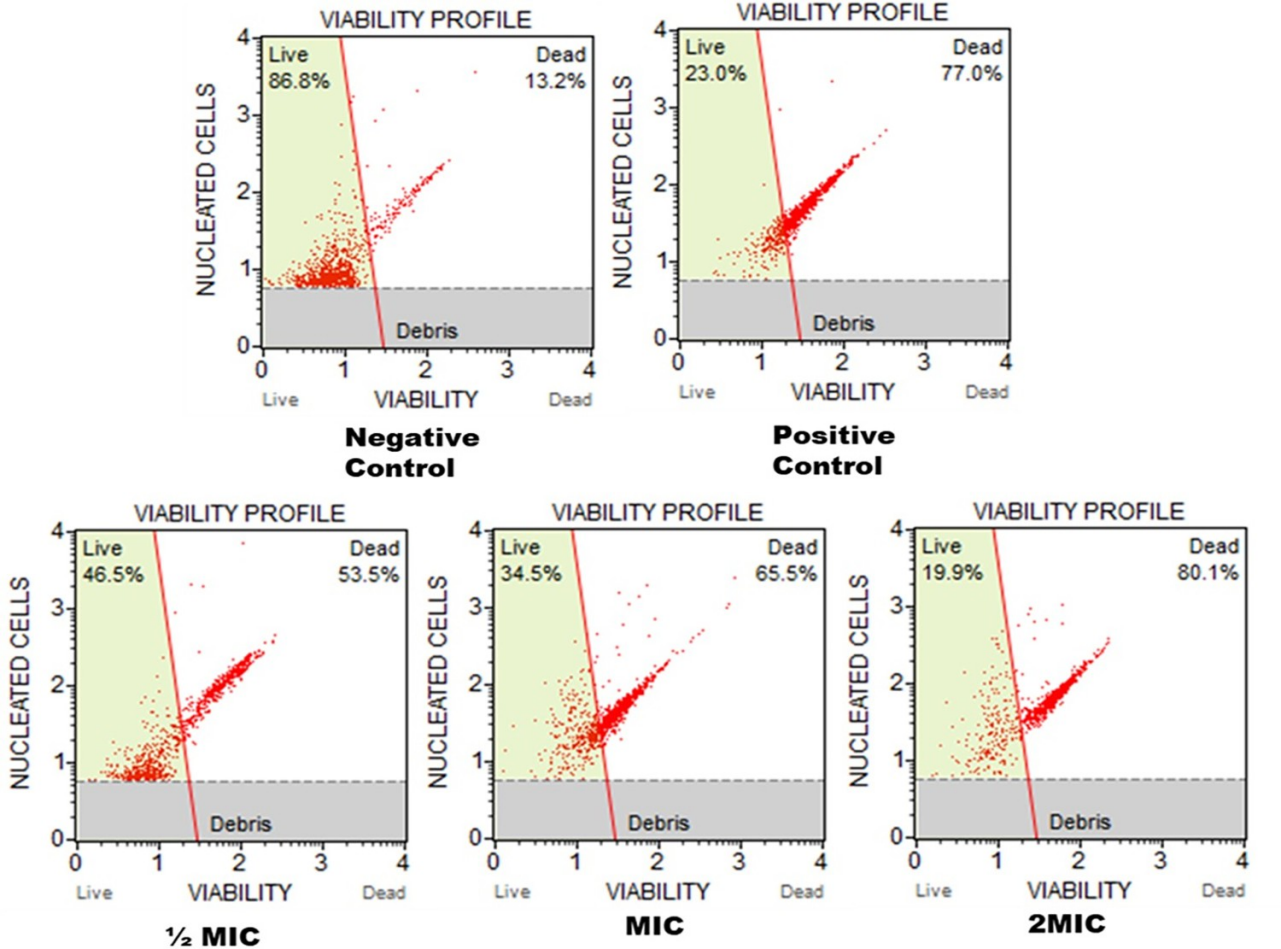
Cell viability of *C*. *auris* 6057. Cell viability of *C*. *auris* 6057 cells was recorded when cells were exposed to ½MIC, MIC and 2MIC of C5 compound. Cells without any exposure and with 10 mM H_2_O_2_ exposed serve as negative and positive controls.

Cell count and viability assay was performed to further demonstrate the susceptibility of *C*. *auris* 6057 cells against the most active compound C5. Fig 2 depicts the cell viability profile and population profile of *C*. *auris* before and after treatment with ½MIC, MIC, and 2MIC concentrations of C5 compound. The percentage (%) of cell viability at ½MIC, MIC, and 2MIC of C5 compound was 46.5%, 34.5%, and 19.9%, respectively (Fig 2). These findings established that the test compound C5 suppresses the growth and viability of *C*. *auris* in a concentration dependent manner and thereby validating the susceptibility results. As expected, negative control contained 86.8% live cells, whereas the positive control contained only 23.0% live cells.

### Cell cycle arrest

**Fig 3:**
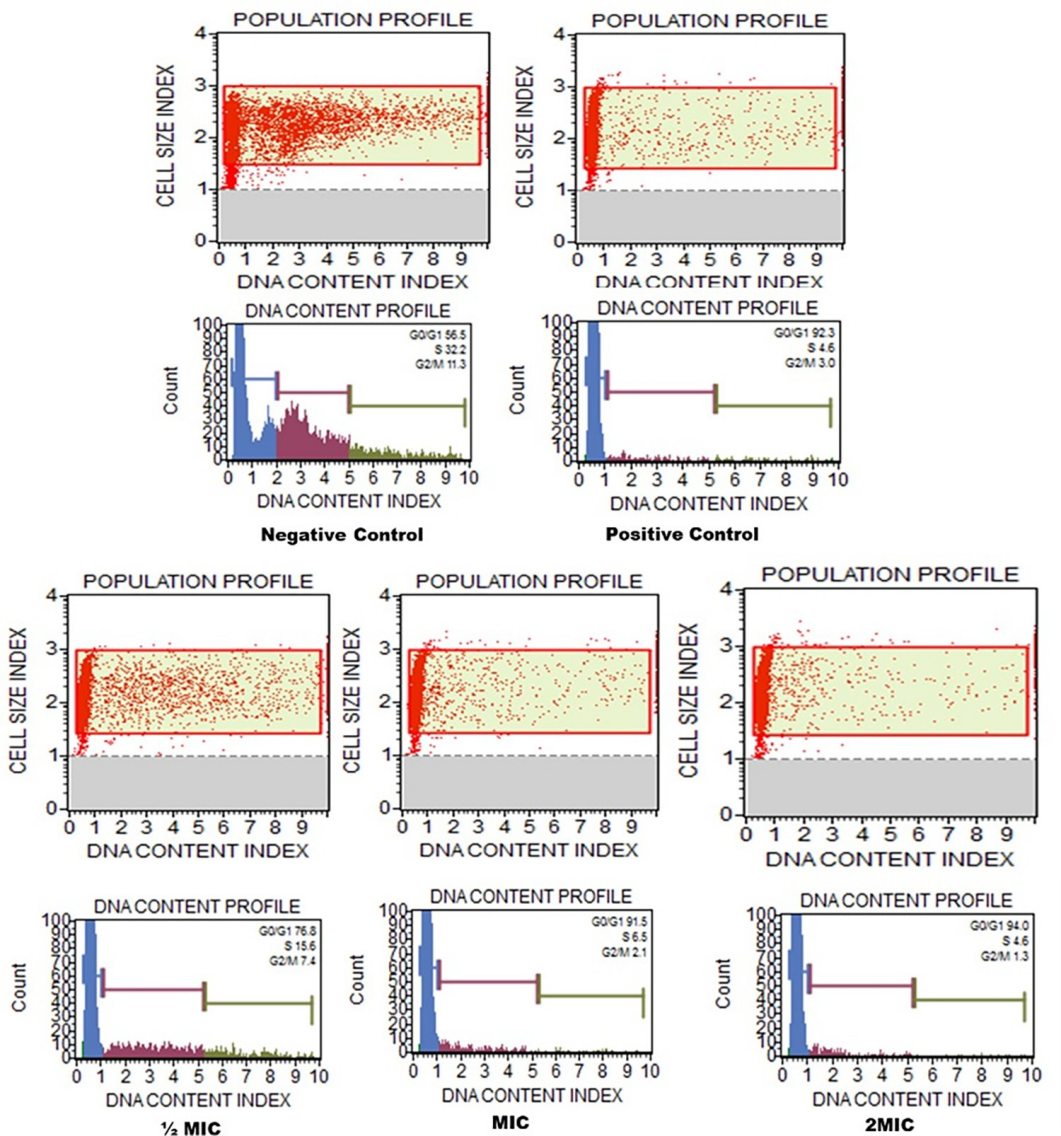
*C*. *auris* cell cycle analysis. The figure shows the effect of compound C5 at ½MIC, MIC and 2MIC on the cycle in *C*. *auris* 6057. Positive controls were cells exposed to H_2_O_2_, and negative controls were healthy untreated *C*. *auris* cells.

Following the susceptibility assays, effect of the C5 compound on the cell cycle of *C*. *auris* 6057 cells was determined using MUSE cell analyzer. The percentage of cells distributed among the various stages of the cell cycle in the untreated cells represent the normally developing cells, while as in comparison cells stuck in one phase indicate cell cycle arrest. The cell cycle results of *C*. *auris* revealed that healthy cells were uniformly distributed with 56% in G0/G1 phase, 32% in S phase and 11% in G2/M phase. In contrast cells treated with C5 halted in G0/G1, and the cell growth was predominantly limited to G0/G1 phase 76.8%, 91.5%, and 94.0% after the exposer with ½MIC, MIC and 2MIC respectively, while as only 15.6%, 6.5% and 4.6% of cells were arrested in S phase when exposed with ½MIC, MIC and 2MIC respectively (Fig 3). In the positive control where cells were treated with H_2_O_2_, then most of the cells (92.3%) were seen arrested in G0/G1 phase and only 4.6% and 3% are arrested in S and G2/M phases respectively.

### Apoptotic studies

Apoptosis is an evolutionarily conserved mechanism used in the regulation of growth and differentiation by all multicellular organisms [28]. Apoptosis and cell cycle are linked to each other and are important in cell survival, proliferation, and reproduction. Arrest of *C*. *auris* cells in G1 phase of cell cycle as seen above could be related to the apoptosis in these cells and thereby their inability to enter S phase. Therefore, the present study determined the effect of C-5 compound against different apoptotic markers in *C*. *auris* 6057.

### Mitochondrial membrane potential (MMP) (Δψm)

**Fig 4:**
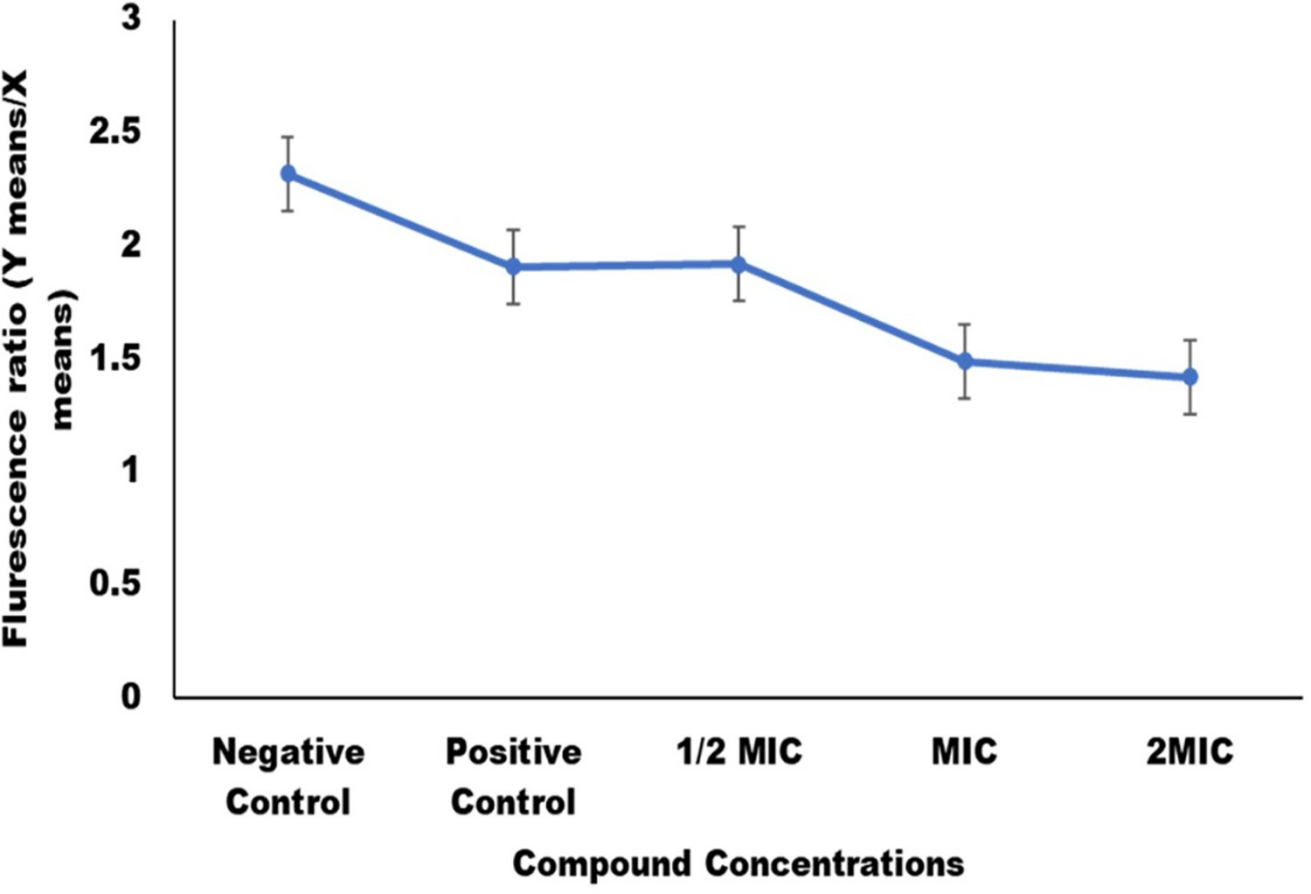
Mitochondrial depolarization in *C*. *auris*. Fluorescence ratio (Y mean/X mean) representing Δψm and thereby apoptosis. In comparison to the negative untreated cells, *C*. *auris* exposed cells with ½MIC, MIC and 2MIC of C-5 compound showed depolarization of the mitochondrial membrane. Positive control represents *C*. *auris* 6057 cells treated with H_2_O_2_.

The membrane potential in mitochondria (Δψm) is an indicator of mitochondrial energetic status. MMP (Δψm) is used to assess the activity of proton-pumps in mitochondria, electron transport systems (ETS) and the activation of mitochondrial permeability due to a variety of causes. Loss of MMP is regarded as very crucial for the survival and death of cells and is necessary stage for the apoptotic cascade. Therefore, C-5 compound was studied for its effect on MMP in *C*. *auris* cells to determine its ability to cause apoptosis. The constant Δψm in living yeast cells allowed the JC-10 to dye to clump together which can be detected by red fluoresce. In contrast apoptotic cells show decreasing Δψm, which restricts the JC-10 dye to monomeric form and thereby resulting in green fluorescence. Δψm was measured by calculating the ratio of JC-10 aggregates to JC-10 monomers; a decrease in values when compared to the untreated control indicated Δψm deprotonation. The results showed that in comparison of untreated cells a significant rise in JC-10 monomer was observed, means fluorescence levels was detected, indicating mitochondrial membrane depolarization (Fig 4). The ratio was 2.32 in the untreated cells or the cells with intact mitochondrial membrane and 1.91 in the positive control cells. In terms of C-5 compound exposure, on increasing the concentration of the compound (½MIC, MIC, and 2MIC) membrane potential decreases from 1.92, 1.49, and 1.42 respectively. These results suggested that the C-5 compound causes membrane disintegration in mitochondria by lowering the mitochondrial membrane potential in *C*. *auris* cells. Mitochondrial membrane depolarization is caused by uncontrolled mitochondrial membrane pores, which induces movement and the activation of various pro-apoptotic proteins [29]. Reactive oxygen species (ROS) that are produced by mitochondria were also recognized to play role in causing apoptosis [30].

### Cytochrome c oxidase activity

**Fig 5:**
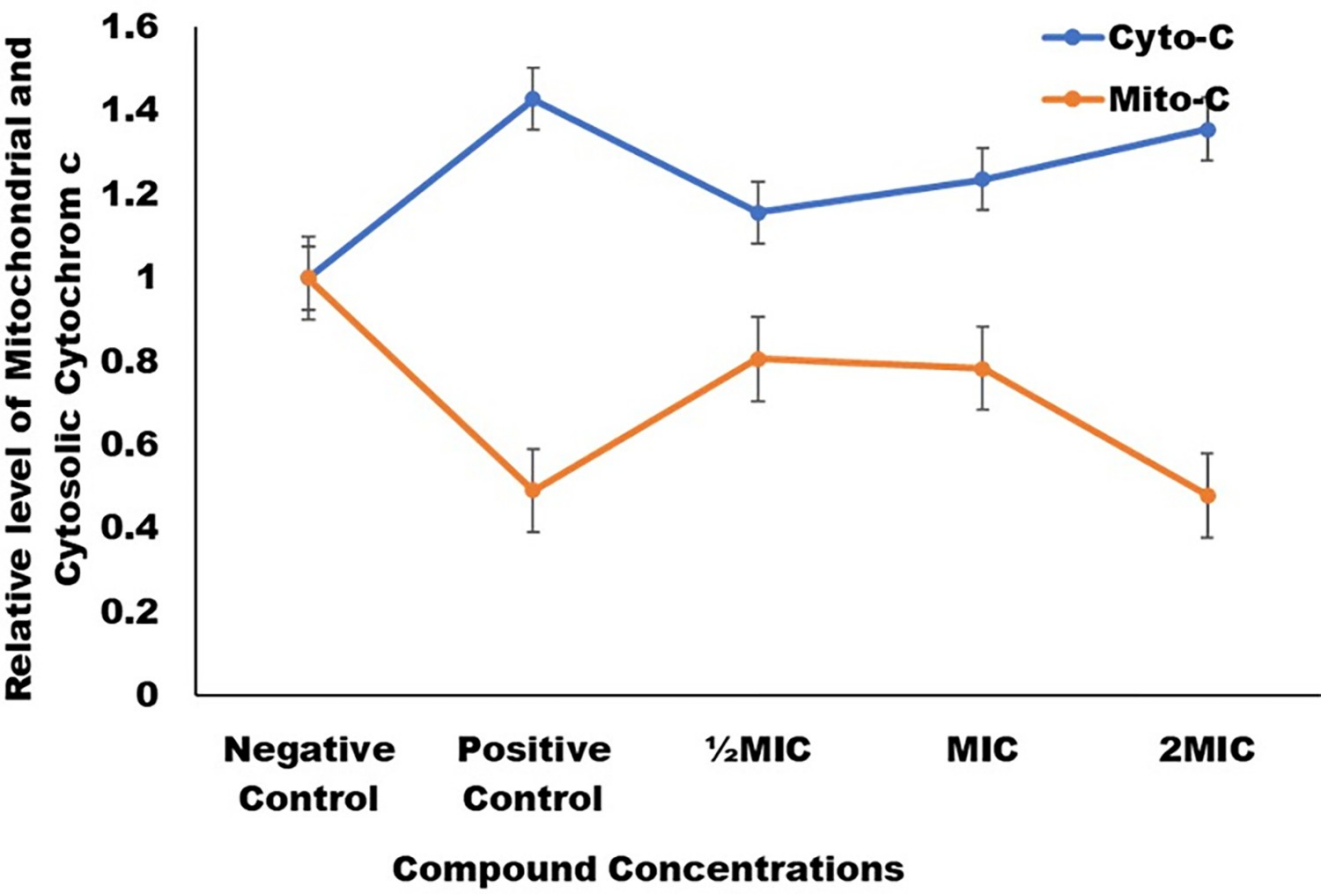
Cytochrome c movement in *C*. *auris*. Movement of apoptotic factor cytochrome c from mitochondria to cytosol in *C*. *auris* 6057 in absence (negative control), H_2_O_2_ treated (positive control) and C-5 treated at ½MIC, MIC and 2MIC. At 550nm, cytochrome c level in the mitochondria (orange line) and the cytosol (blue line) were calculated.

Determination of cytochrome c oxidase activity is an established method for studying apoptotic cell death. This quantitative method involves the measurement of the amount of cytochrome c oozing out from mitochondria to cytosol. This study examined the changes in cytochrome c levels in mitochondria and cytosol in untreated and treated cells with various concentrations of compound C-5. According to the observations, there was a considerable increase of cytochrome c level in cytosol and decrease in mitochondrial cytosol when cells were treated with C-5 in comparison to untreated cells (Fig 5). Untreated negative control cells had relative fluorescence values which were consider 1.0 for cytochrome c in both mitochondria and cytosol. In comparison, congener C-5 treatment caused significant ooze out of cytochrome c in *C*. *auris* cells at ½MIC, MIC, and 2MIC values, with relative fluorescence values of 0.8, 0.78 and 0.47 for mitochondrial and 1.15, 1.24 and 1.35 for cytosolic cytochrome c, correspondingly (Fig 5). The cytochrome c in positive control was 0.49 for mitochondrial cytochrome c and 1.43 for cytosolic cytochrome c. Cytochrome c is a component of the electron transport chain (ETC) that is loosely connected to the inner mitochondrial membrane. Caspases are activated by the release of cytochrome c from the mitochondria to the cytoplasm, which is a critical step in the induction of apoptotic cell death [18].

### DNA damage by TUNEL assay

**Fig 6:**
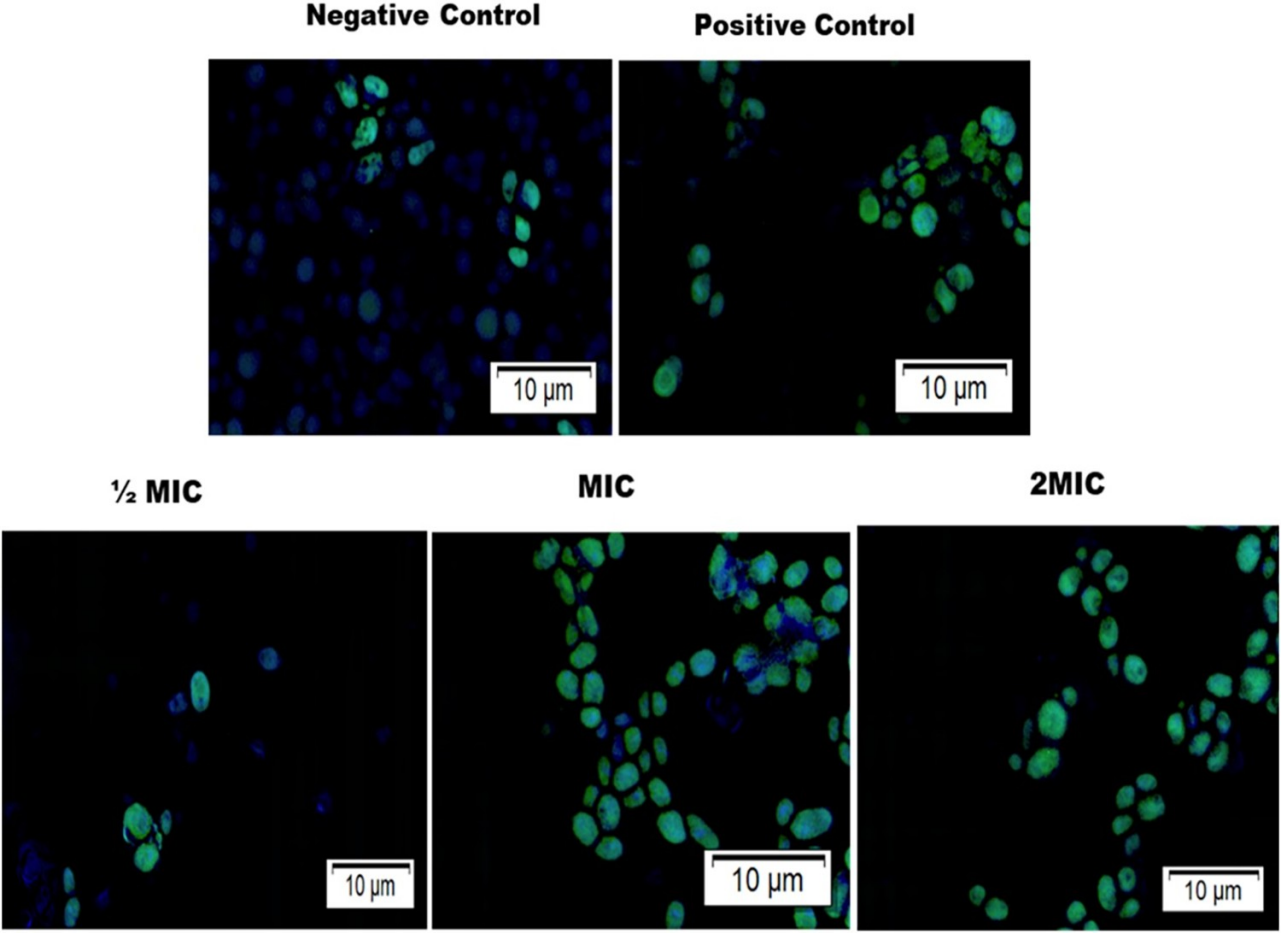
TUNNEL assay representing apoptosis in *C*. *auris*. The confocal microscopy of *C*. *auris* 6057 cells treated with different concentrations (½MIC, MIC, and 2MIC) of C-5 compound. Hoechst 33342 dye (blue fluorescence) showing live cells, and Alexa Fluor 488 dye (green fluorescence) represent apoptotic cells.

The phosphatidylserine externalization is another important parameter for apoptosis studies. The terminal deoxynucleotidyl transferase-mediated dUTP nick end labeling (TUNEL) test was used to investigate this hallmark of apoptosis in response to the active drugs. The TUNEL-assay is based on the incorporation of modified dUTP at the 3′-OH ends of fragmented DNA. *C*. *auris* cells were additionally stained with a Hoechst 33342 dye for differentiation, as it produces blue color for live cells. From the results, *C*. *auris* cells exposed to various concentrations (½MIC, MIC, and 2MIC) of the compound C-5, showed considerable increase in the amount of TUNEL positive nuclei (green color fluorescent spots) when compared to the unexposed cell population **(**Fig 6).

The results are suggesting the dose dependent effect, with higher concentrations of the test compound C-5 by increasing the population and intensity of green fluorescence cells in TUNEL positive nuclei. Positive control (10 mM H_2_O_2_ treated) also showed enhanced green fluorescence, showing a higher population of late apoptotic cells, in comparison of negative control with more blue fluorescence live cell population.

### Cytotoxicity

**Fig 7:**
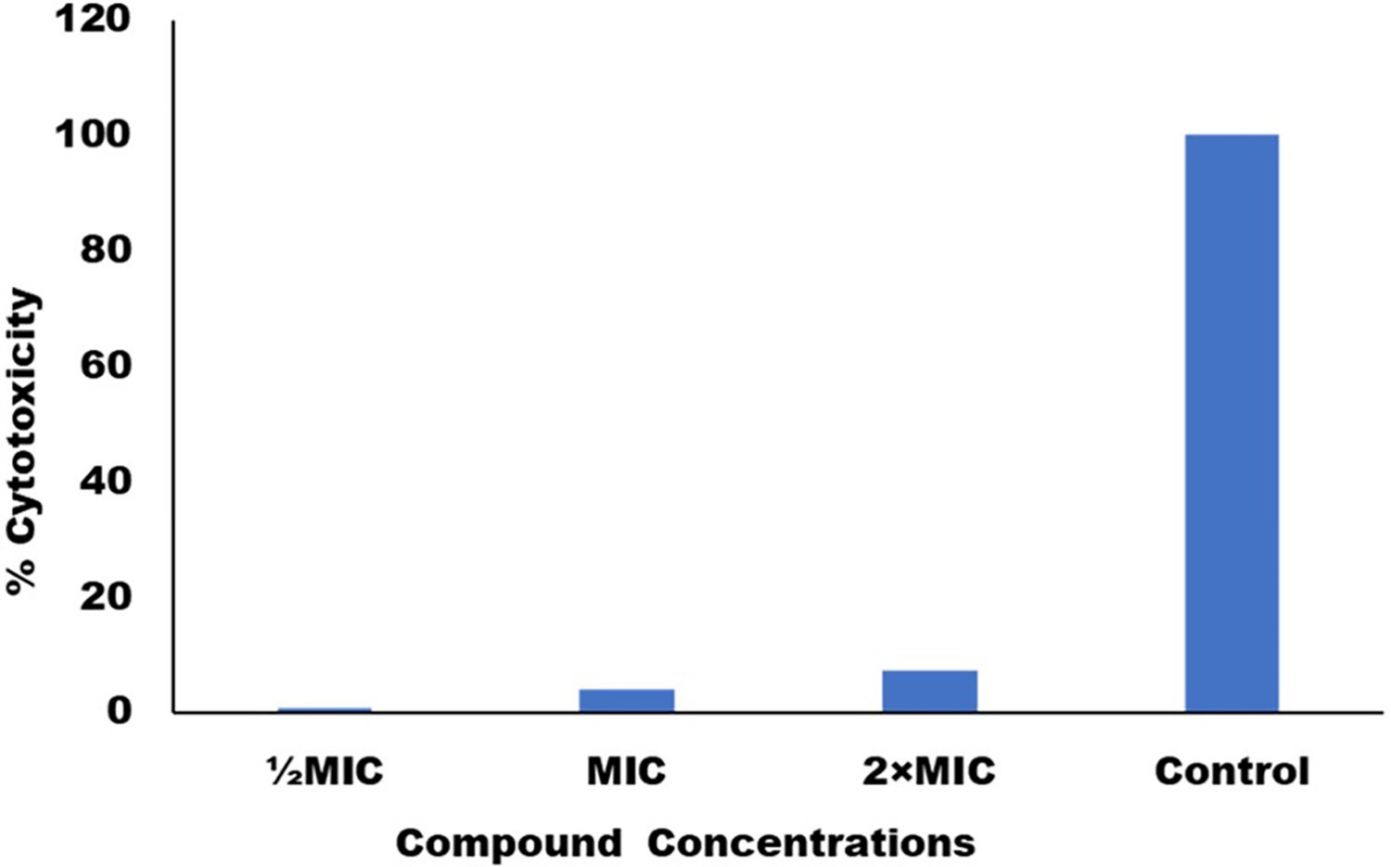
Hemolytic activity of C-5. Hemolysis of horse red blood cells was done in presence of Triton-X (control) and different concentrations of C-5.

The cytotoxicity of C-5 at various doses ½MIC, MIC, and 2MIC was tested against horse blood cells (RBCs). We corelate the Triton X-100 as a positive control, which induces 100% RBSs lysis, test compound C-5 caused hemolysis 0.6%, 3.92%, and 7.3% at ½MIC, MIC, and 2MIC values (Fig 7). PBS was utilized as a negative control, and no RBCs were lysed. The results confirmed that the newly synthesized C-5 compound has lower cytotoxicity, implying that it is potentially safe to use for in-vivo studies and thus provide a potential candidate for antifungal drug development.

### Conclusion

Significant antifungal activity caused by apoptosis and cell cycle arrest in *C*. *auris* by one of the eugenol derivatives (C5) was observed in this study. This compound showed potent antifungal activity against the tested *C*. *auris* isolates, and therefore shows promise as a lead molecule that could be studied further. Furthermore, unlike eugenol this lead compound also displayed low toxicity against red blood cells urging its safe use for *in vivo* assays. To put together, these results are quite encouraging and therefore further detailed studies of the active compound against different fungal pathogens would reveal more about the potential of this compound as a lead molecule.

### Statistical analysis

All experiments were performed independently in triplicates (n = 3), and data were presented as mean ± standard deviation (SD). The statistical analysis was performed using two-way ANOVA test by GraphPad Prism software, version 8.0.1.

## Supporting information

S1 File Supporting information contains ^1^H and ^13^CNMR data of the compounds C1-C6, and Scheme (S1 Scheme) for the synthesis of the compounds. (DOCX)
