# Supplementary material for: Cellular apoptosis and cell cycle arrest as potential therapeutic targets for eugenol derivatives in Candida auris
Source: PLoS One. 2023 Jun 21;18(6):e0285473. doi: 10.1371/journal.pone.0285473 (PMC10284410; doi:10.1371/journal.pone.0285473)
Supplement: S1 File — (DOCX) [file pone.0285473.s001.docx]

**SUPPORTING INFORMATION**

**Cellular apoptosis and cell cycle arrest as potential therapeutic targets for eugenol derivatives in *Candida auris***

**Hammad Alam^1^, Vartika Shrivastav^1^, Windy Sekgele^2^, Mohmmad Younus Wani^3*^, Abdullah Saad Al-Bogami^3^, Julitha Molepo^2*^, Aijaz Ahmad^1,4^**

*^1^Department of Clinical Microbiology and Infectious Diseases, School of Pathology, Faculty of Health Sciences, University of the Witwatersrand, Johannesburg, 2193, South Africa*

*^2^Department of Oral Biological Sciences, School of Oral Health Sciences, Faculty of Health Sciences, University of the Witwatersrand, Johannesburg, 2193, South Africa*

*^3^Department of Chemistry, College of Science, University of Jeddah, Jeddah 21589, Saudi Arabia*

*^4^Infection Control, Charlotte Maxeke Johannesburg Academic Hospital National Health Laboratory Service, Johannesburg, 2193, South Africa.*

^*^Author for correspondence: [mwani@uj.edu.sa](mailto:mwani@uj.edu.sa) (MYW); [Julitha.Molepo@wits.ac.za](mailto:Julitha.Molepo@wits.ac.za) (JM)

**^1^HNMR and ^13^C NMR of derivatives C1-C6**


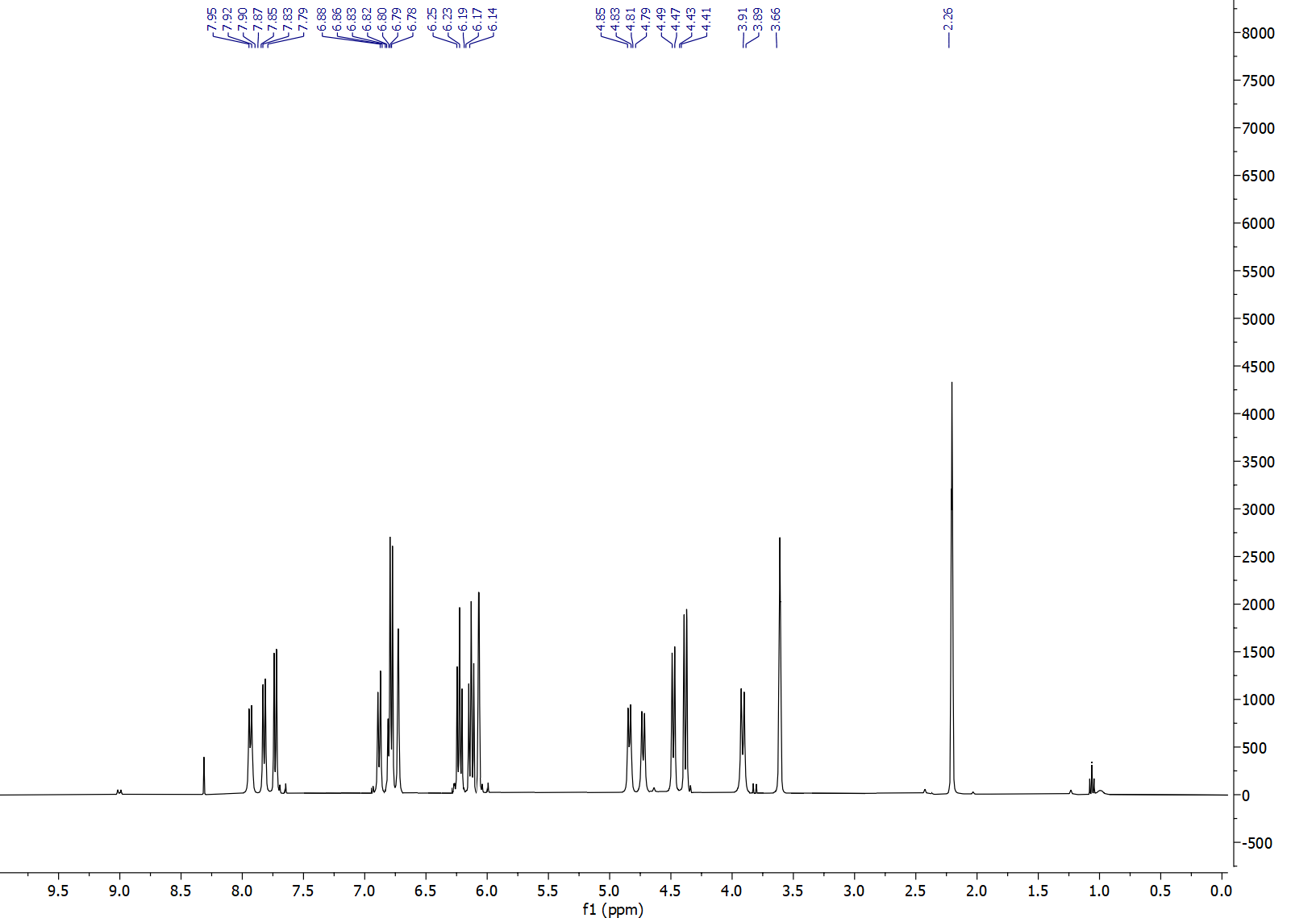


**^1^HNMR_C1**


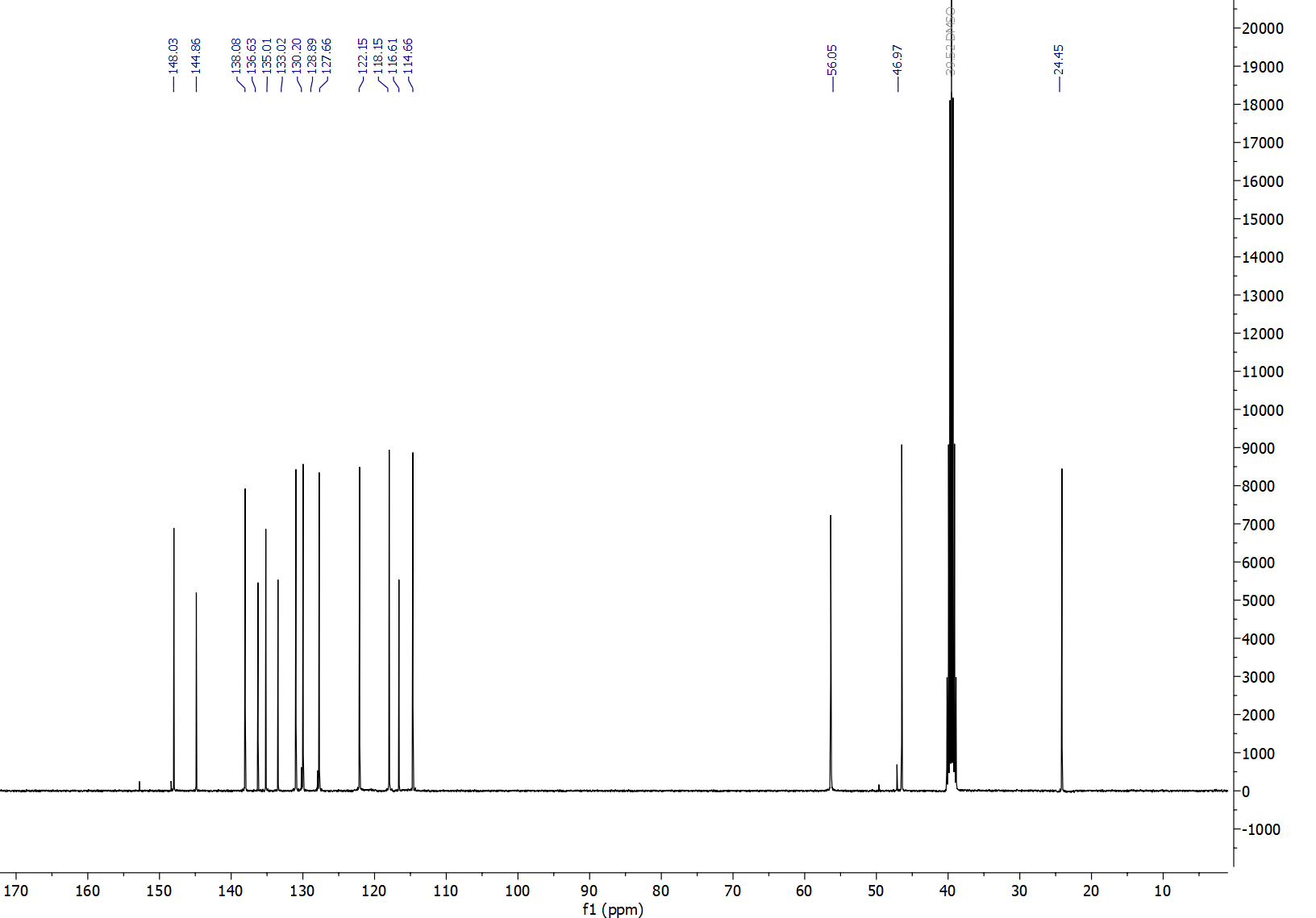


**^13^CNMR_C1**


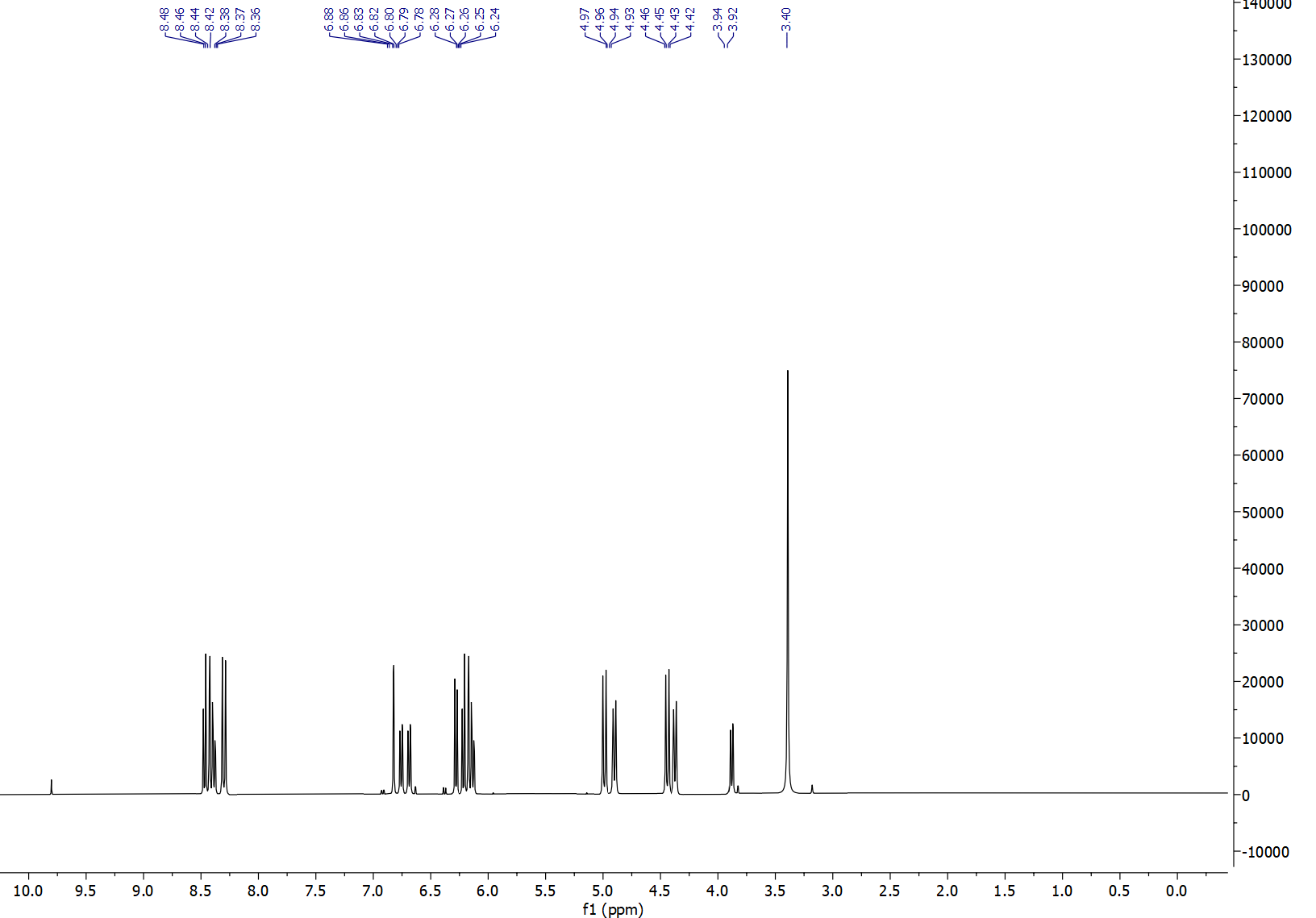


**^1^HNMR_C2**


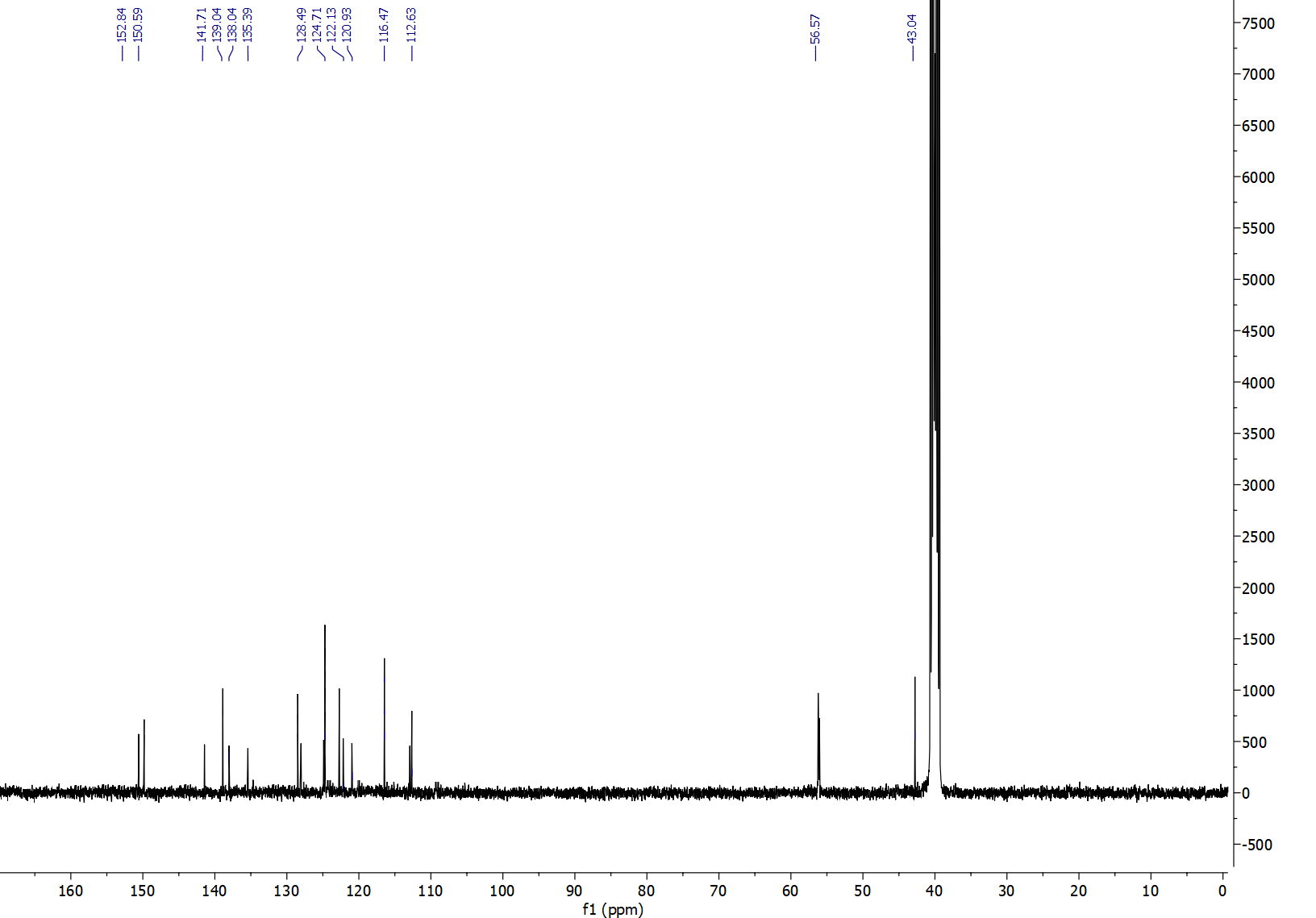


**^13^CNMR_C2**


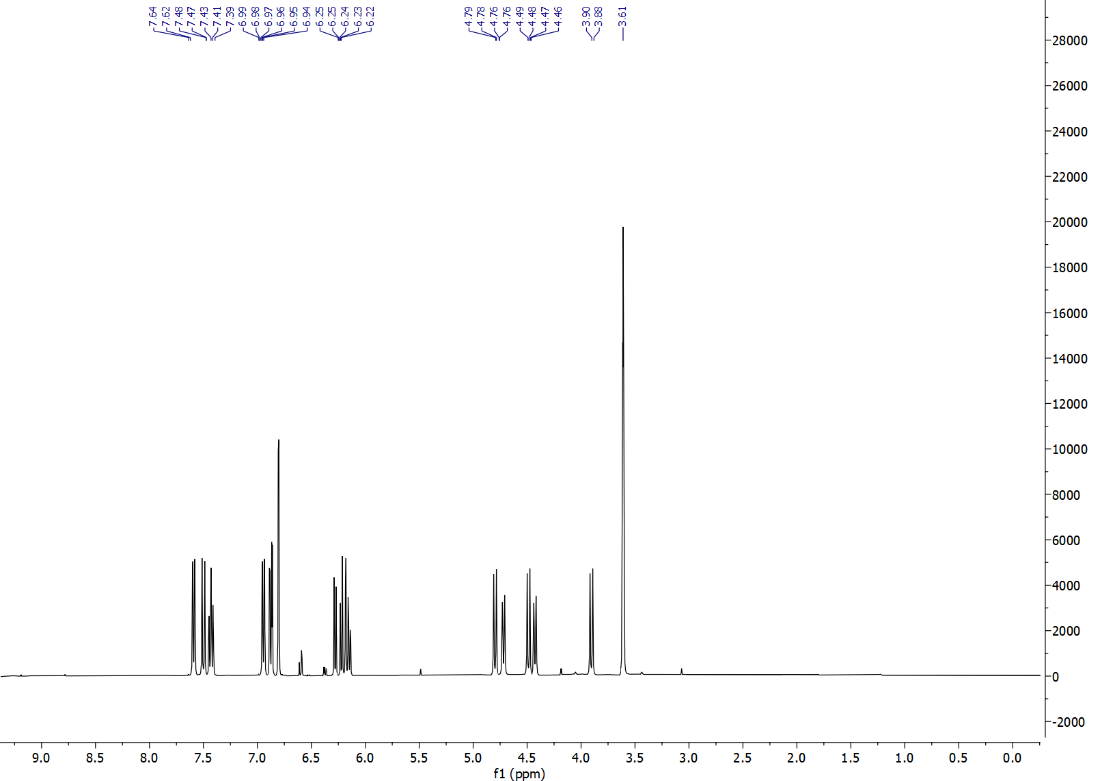


**^1^HNMR_C3**


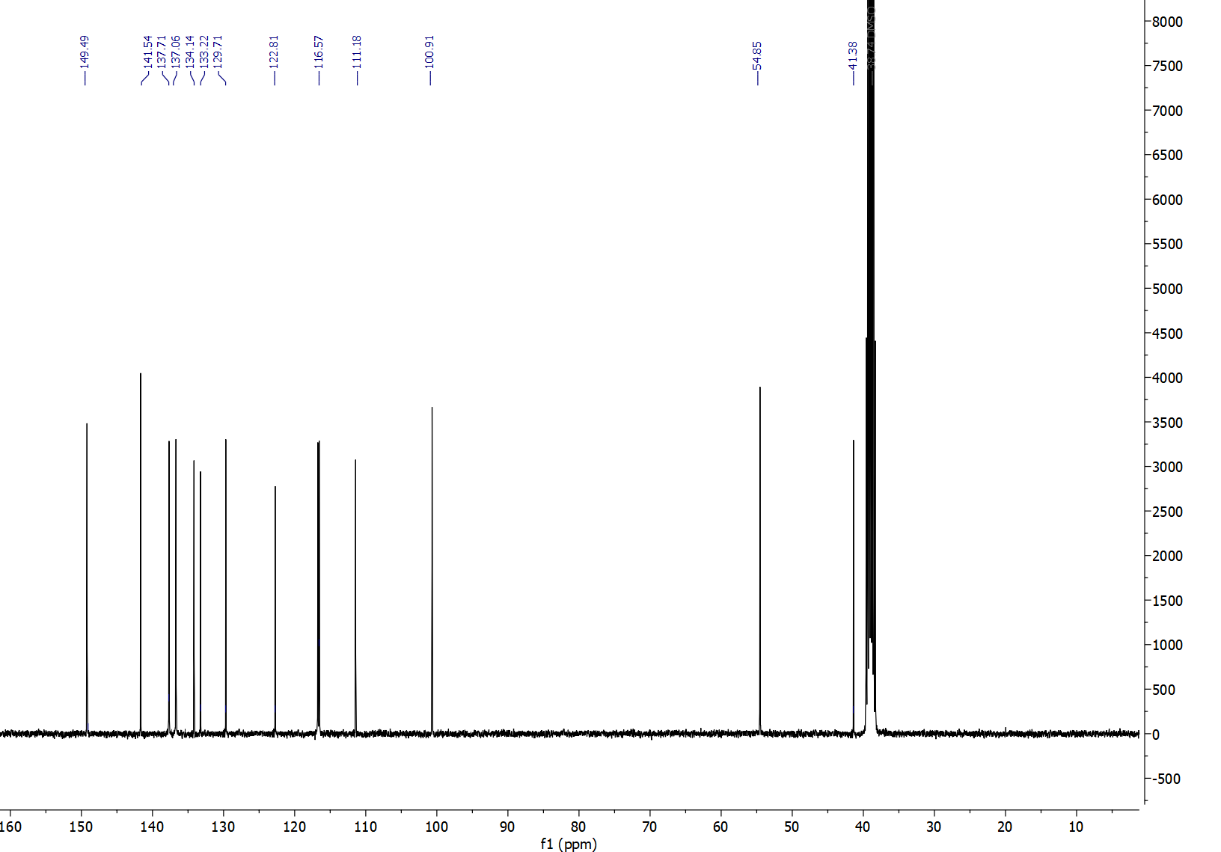


**^13^CNMR_C3**


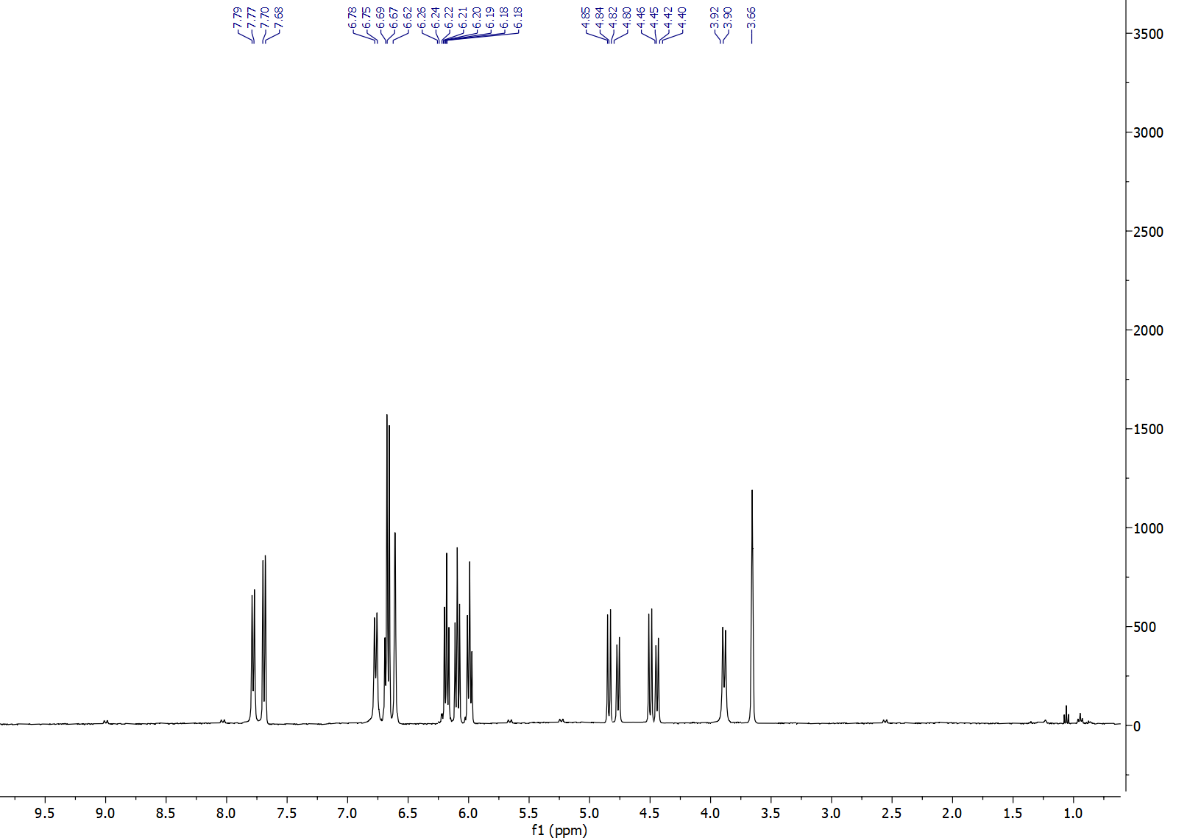


**^1^HNMR_C4**


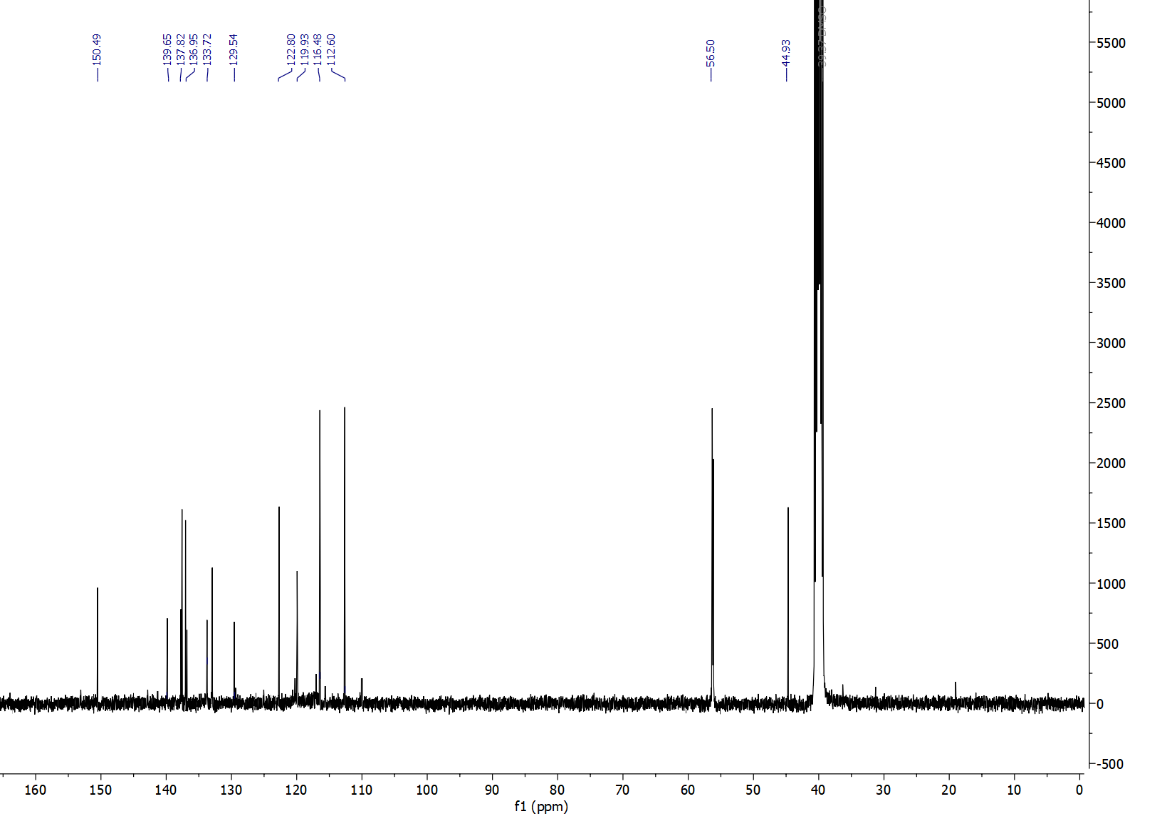


**^13^CNMR_C4**


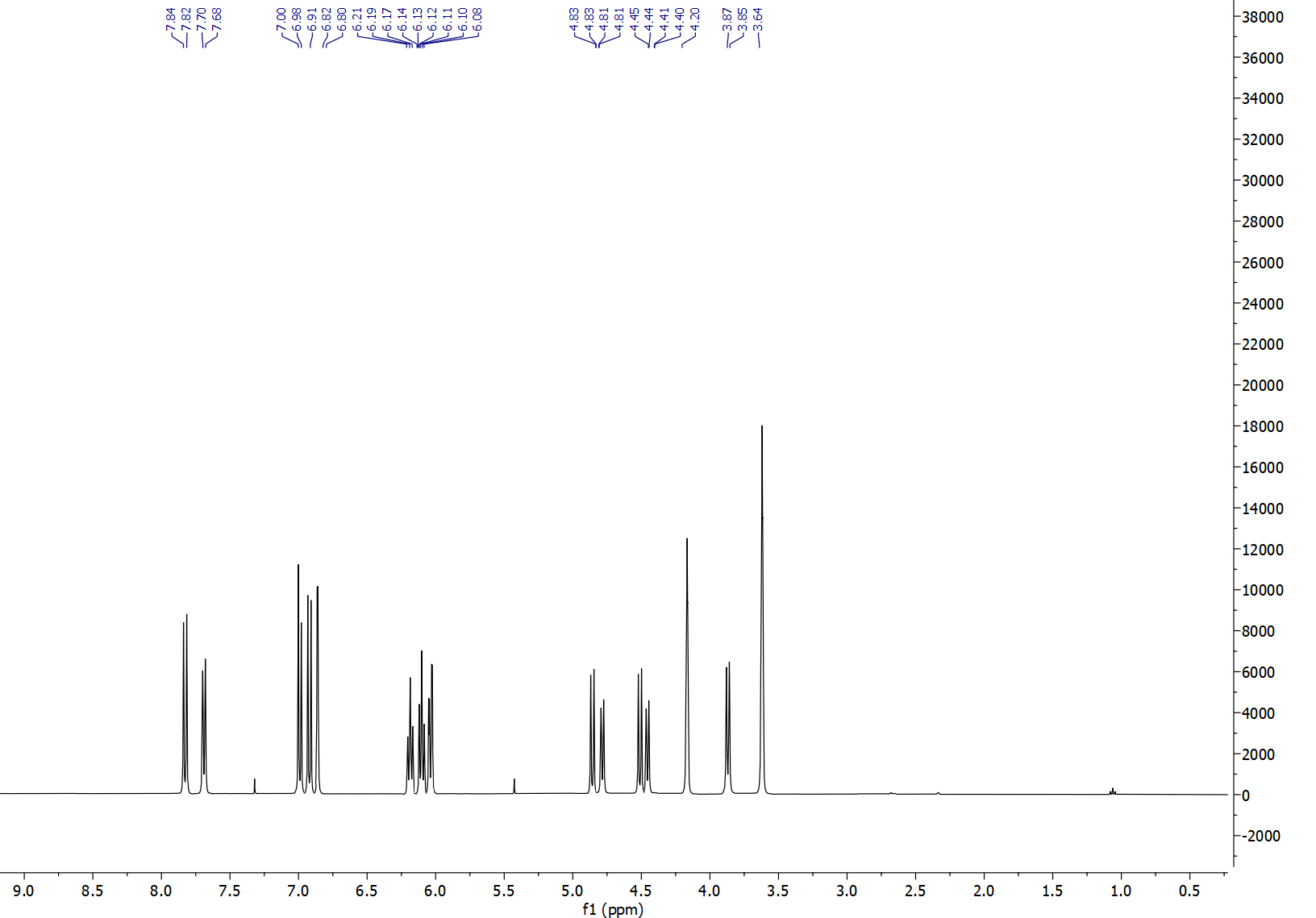


**^1^HNMR_C5**


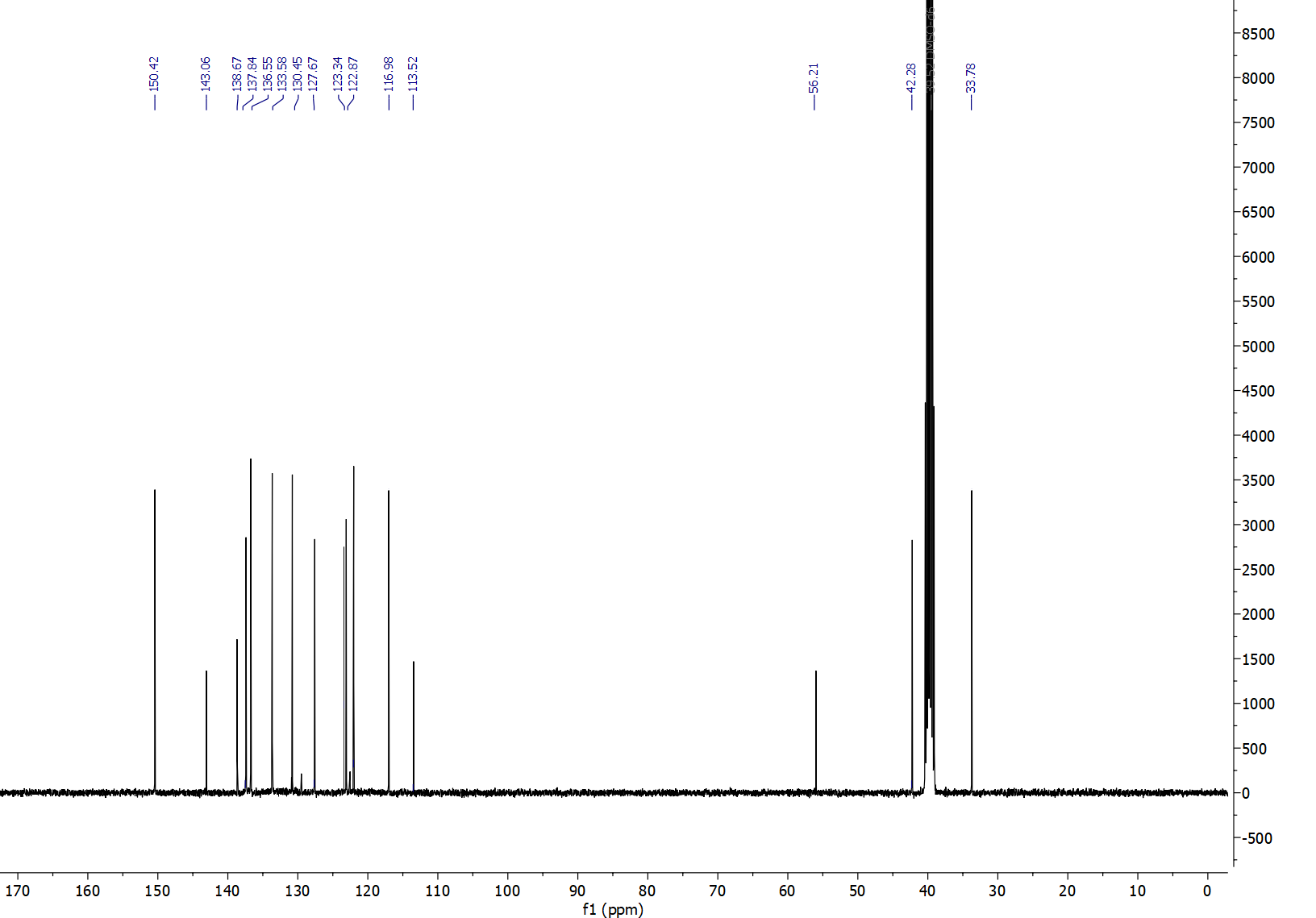


**^13^CNMR_C5**


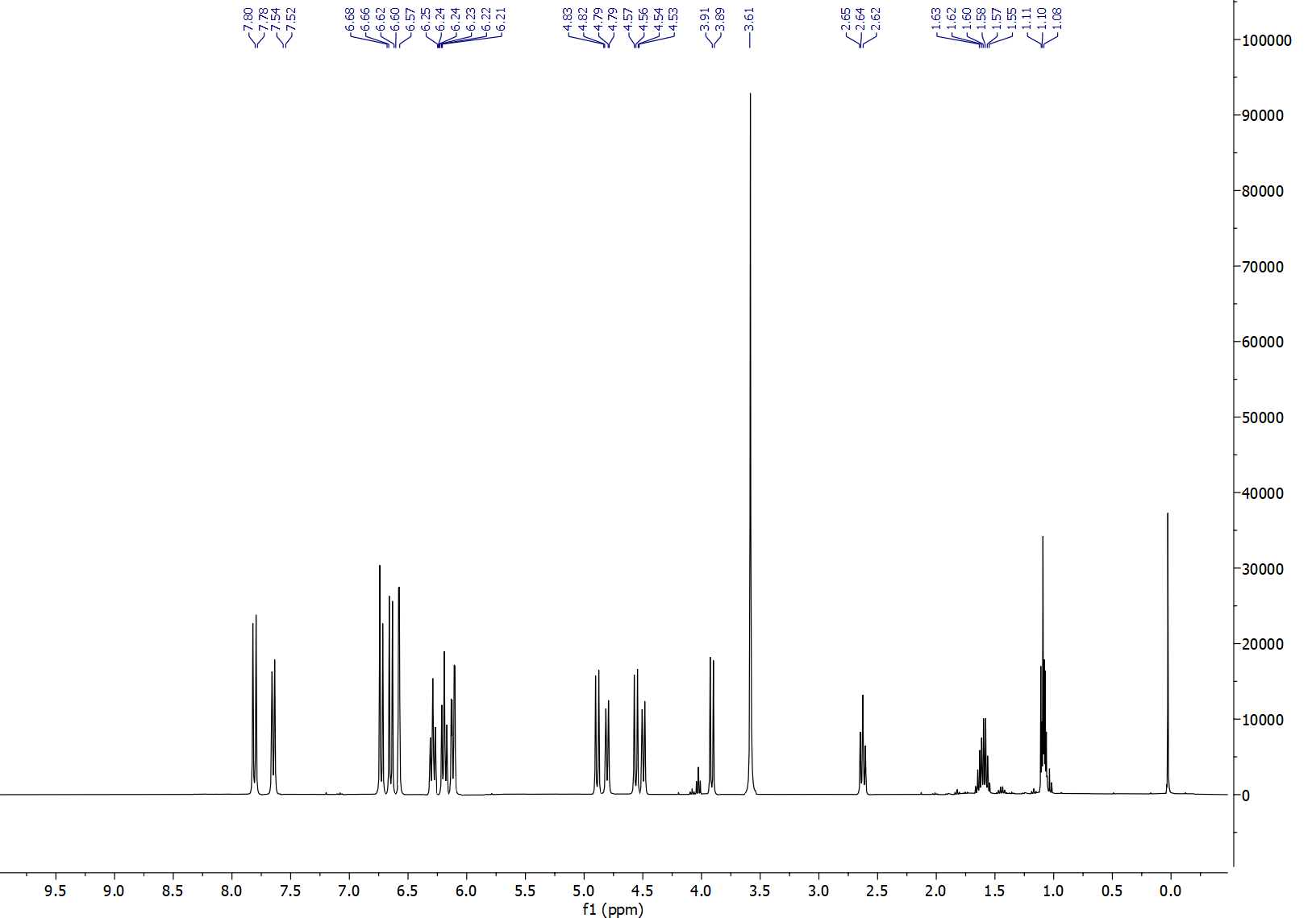


**^1^HNMR_C6**


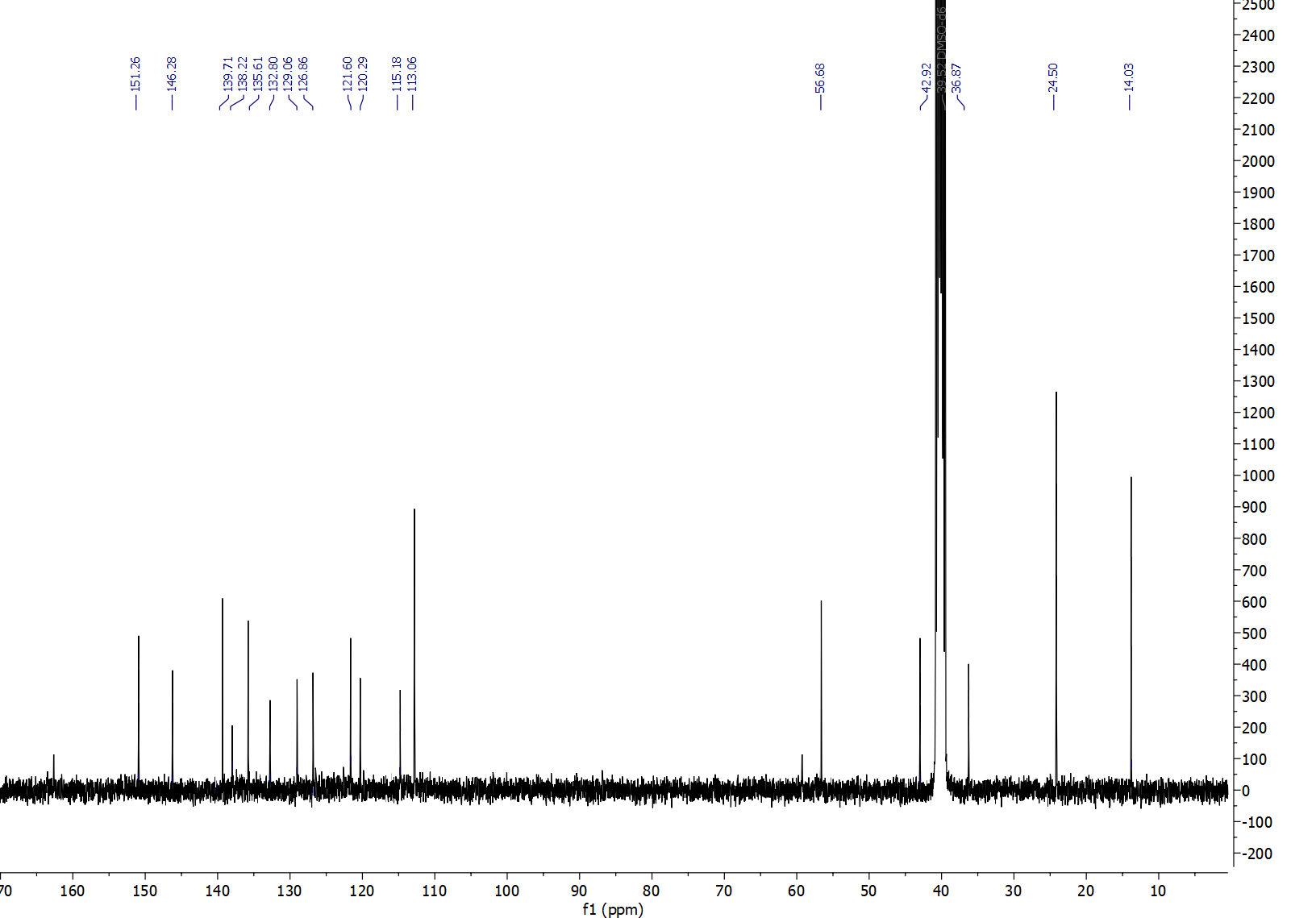


**^13^CNMR_C6**

**Scheme S1:** Synthesis of eugenol tosylate congeners **C1-C6**; *Reaction conditions: sulfonylchlorides, pyridine, reflux 18-24h*
